# Patterns of Skills Review in Smartphone Cognitive Behavioral Therapy for Depression: Observational Study of Intervention Content Use

**DOI:** 10.2196/63497

**Published:** 2025-02-24

**Authors:** Emily E Bernstein, Katharine E Daniel, Peyton E Miyares, Susanne S Hoeppner, Kate H Bentley, Ivar Snorrason, Lauren B Fisher, Jennifer L Greenberg, Hilary Weingarden, Oliver Harrison, Sabine Wilhelm

**Affiliations:** 1 Massachusetts General Hospital Boston, MA United States; 2 Harvard Medical School Boston, MA United States; 3 University of Virginia Charlottesville United States; 4 HabitAware, Inc Minneapolis, MN United States; 5 Koa Health London United Kingdom

**Keywords:** smartphone, cognitive behavioral therapy, engagement, depression, mental health, Mindset, mHealth, mobile health, app, digital health, mobile phone

## Abstract

**Background:**

Smartphones could enhance access to effective cognitive behavioral therapy (CBT). Users may frequently and flexibly access bite-size CBT content on personal devices, review and practice skills, and thereby achieve better outcomes.

**Objective:**

We explored the distribution of actual interactions participants had with therapeutic content in a trial of smartphone CBT for depression and whether interactions were within assigned treatment modules or revisits to prior module content (ie, between-module interactions).

**Methods:**

We examined the association between the number of within- and between-module interactions and baseline and end-of-treatment symptom severity during an 8-week, single-arm open trial of a therapist-guided CBT for depression mobile health app.

**Results:**

Interactions were more frequent early in treatment and modestly declined in later stages. Within modules, most participants consistently made more interactions than required to progress to the next module and tended to return to all types of content rather than focus on 1 skill. By contrast, only 15 of 26 participants ever revisited prior module content (median number of revisits=1, mode=0, IQR 0-4). More revisits were associated with more severe end-of-treatment symptom severity after controlling for pretreatment symptom severity (*P*<.05).

**Conclusions:**

The results suggest that the frequency of use is an insufficient metric of engagement, lacking the nuance of what users are engaging with and when during treatment. This lens is essential for developing personalized recommendations and yielding better treatment outcomes.

**Trial Registration:**

ClinicalTrials.gov NCT05386329; https://clinicaltrials.gov/study/NCT05386329?term=NCT05386329

## Introduction

An estimated 21 million US adults experienced at least 1 major depressive episode in 2021; yet, only 61% of these individuals are expected to have received any past-year treatment [1]. Mental health care providers caution that waitlists already far exceed what can be addressed by the current workforce and demands for treatment are expected to continue to rise [2]. Smartphone-delivered psychotherapies offer increased access to care in the face of limited clinician availability by reducing clinician hours or duration of treatment. Promisingly, cognitive behavioral therapy (CBT) treatments delivered fully or in part via smartphone apps have demonstrated efficacy for depression [3-5].

App-delivered CBT can be implemented as a standalone, self-guided treatment or alongside varying degrees of human support (eg, asynchronous messaging or teletherapy sessions). Apps can ideally offer users “bite-size,” high-quality psychoeducation and empirically supported treatment tools that can be unobtrusively accessed on their personal smartphone when and where it would be most helpful. For example, a user might return to an earlier lesson on cognitive restructuring for a quick reminder about the skill when feeling upset at work; later that day, they might open their ongoing activity scheduling log when feeling unmotivated. By serving as an on-demand “pocket therapist,” app-delivered CBT might even stand to improve treatment response rates, which are only around 42% for face-to-face CBT for major depressive disorder (MDD; see [6] for meta-analytic review). Indeed, a core tenet across CBTs is that reviewing and practicing skills is necessary for recipients to benefit and see long-term gains; this effect has been shown in traditional face-to-face formats as well as single-session interventions and digital formats [7-11]. The CBT model aims to equip patients with the ability to recognize patterns in their own thoughts, feelings, and behaviors and routinely apply new approaches to emotional situations. To do so requires repetition; at minimum, identifying a pattern necessitates multiple data points, and new thought or behavioral patterns must replace old ones [12]. Thus, the unique benefit of flexible, real-world treatment integration may depend on users engaging with app content frequently and accessing a range of different skills that are well-matched to their needs.

In reality, however, app-based treatments often struggle with high user dropout [13,14] and little is known about how users actually interact with app content throughout treatment [15]. Indeed, app-delivered interventions for depression are commonly completed by fewer than 50% of research participants [16] and 1 study of high-fidelity smartphone CBT treatments showed the modal number of sessions is one [17]. These sobering findings undermine the assumption that providing treatment material at one’s fingertips will translate into frequent use of that material.

Of those who stay in app-supported treatments, questions remain regarding the dose (both per access and frequency of access) needed for clinical benefit [16]. In general, meta-analysis suggests that greater engagement with digital interventions is associated with improvement in mental health symptoms [18]. However, this conclusion is complicated by the fact that operationalizations for app engagement vary widely (ie, time spent on an app, number of messages sent, number of logins, number of modules completed, frequency of self-monitoring, days used, and self-reported frequency of use) [19,20] as do strength and direction of results. In this vein, some work suggests that the relationship between app use and depression treatment outcomes is nonlinear [21], which further contradicts the belief that users who engage more with app content will necessarily benefit most.

CBT models assume that by building up a person’s repertoire of skills, they will be better able to handle the diverse challenges faced in life [22]. It is perhaps unsurprising, then, that one systematic review found that higher module completion rates—more so than frequency of app use—are associated with better depression outcomes [23]. Thus, the nonlinear relationship between the frequency of app use and outcomes may suggest that differences in how a user engages with app content (what content they use rather than how often they use the app overall) predict intervention outcomes. It is also possible that building mastery in one or two specific skills (ie, repeating frequently) will be most beneficial [24]. For example, 1 study found that participants who used behavioral activation content within the provided CBT for MDD app were more likely to benefit than participants who did not use this content [25]. Moreover, focusing on content or skills practice solely during the treatment module in which it is introduced or formally assigned may be less beneficial than practicing those skills the same number of times but spaced throughout treatment and thus integrating a greater range of skills. Indeed, data from internet-delivered interventions show wide variability in how much different users look at content within versus across treatment sessions [26], suggesting that similar between-user differences may emerge during app-based interventions. The impact of such heterogeneity remains an open question. More clarity may be gained by homing in on what material or skills users return to and when, rather than treating all bouts of app use as interchangeable. Specifically, we introduce a novel dimension of engagement, differentiating between skills review or practice that occurs within a treatment step (akin to rehearsing what a person learned from their therapist that week) from that which occurs across steps (akin to going back to old lessons or skills to sustain gains or implement in new situations). The latter is particularly compelling given how flexibly treatment developers imagine their tools will be used and the field’s lack of data on how users navigate them in practice.

This study therefore investigates how users accessed therapeutic content throughout an 8-week therapist-guided CBT for depression smartphone treatment [27] to test two predictions: (1) users will access content more than required within modules and will revisit content from previous modules; and (2) more frequent within- and between-module content review will be associated with better outcomes. Specifically, we visualized and quantified the degree to which users accessed various app content both within and across 8 different treatment modules. We then explored whether different use patterns were associated with baseline characteristics and treatment outcomes.

## Methods

### Ethical Considerations

The study was approved by the institutional review board of Massachusetts General Hospital (2020P001958). All participants provided informed consent before the initiation of study procedures and were given the ability to opt out at any point. Data were deidentified to protect participants’ privacy. Participants were compensated US $25 at mid-treatment, end of treatment, and 3-month follow-up assessments.

### Study Design

This is a secondary analysis of a recent trial of Mindset for Depression (Mindset) [27]. Participants completed an 8-week trial of app-led CBT for MDD with therapist support (through brief weekly video-conferencing appointments and asynchronous in-app secure messaging). Assessments used in this project were conducted at baseline (pretreatment) and week 8 (posttreatment). Self-report measures were administered via web-based survey during the aforementioned assessment visits. Study staff monitored for measure completion and were able to confirm with participants whether any measures were left blank intentionally or required completion. App use data were collected continuously via the Mindset app.

### Participants

A total of 28 adults enrolled in an open trial of Mindset [27]. Further, 2 participants completed the trial but were excluded from the present analyses due to a technical issue resulting in missing app use data. Additionally, 2 further participants did not complete the trial and were lost to follow-up; however, as app use data were available for these participants, they were retained in these analyses. This resulted in a final sample of 26 adults. To be included in the trial, individuals were aged at least 18 years, living in Massachusetts, presenting with a current primary *DSM-5* (*Diagnostic and Statistical Manual of Mental Disorders* [*Fifth Edition*]) diagnosis of MDD and at least moderately severe symptoms (Patient Health Questionnaire-9 [PHQ-9] score ≥10; [28]), and, if taking psychotropic medication, were on a stable dose of those medications for at least 2 months before enrollment and throughout the trial. Potential participants were excluded if they had already completed 4 or more prior sessions of CBT for depression; presented with current severe substance use disorder, lifetime bipolar disorder or psychosis, or acute and active suicidal ideation (as indicated by either clinical judgment or a score ≥2 on the past month suicidal ideation subscale of the Columbia-Suicide Severity Rating Scale [29]); were engaged in concurrent psychological treatment; or were unable to engage with treatment (eg, did not own a supported mobile smartphone). See Table 1 for a summary of demographics. Table 2 includes baseline clinical characteristics.

**Table 1:** Baseline demographic features of the sample (N=26).

Characteristic		Sample
Age (years), mean (SD)		33.69 (11.22)
**Gender, n (%)**		
	Woman	21 (80.77)
	Man	5 (19.23)
**Sexual orientation, n (%)**		
	Bisexual	5 (19.23)
	Gay, lesbian, or homosexual	1 (3.85)
	Straight or heterosexual	18 (69.23)
	Some other sexual orientation	2 (7.69)
**Race, n (%)**		
	American Indian, Alaskan Native, or First Nations	1 (3.85)
	Asian	3 (11.54)
	Black or African American	1 (3.85)
	White	18 (69.23)
	More than one race	2 (7.69)
	Some other race	1 (3.85)
**Ethnicity, n (%)**		
	Hispanic or Latino	5 (19.23)
	Not Hispanic or Latino	21 (80.77)

**Table 2:** Spearman correlations among app interactions and clinical features.

Variable	Mean	IQR^a^	ω_t_^b^	1	2	3	4	5	6	7	8	9	10
1. Number of total interactions	71.54	49-96	N/A^c^										
2. Number of between-module interactions	4.08	0-4	N/A	0.27									
3. Number of within-module interactions	67.46	49-80	N/A	0.98^***^	0.16								
4. Baseline HAM-D^d^	19.54	17-23	0.77	–0.19	–0.2	–0.23							
5. Baseline PHQ-9^e^	15.35	12-18	0.83	0.18	0.27	0.11	0.49^*^						
6. Baseline treatment credibility	18.88	17-20	0.68	–0.05	0.32	–0.11	0	–0.09					
7. Baseline treatment expectancy	13.97	11.8-16.6	0.84	0.03	0.27	0.04	–0.07	–0.22	0.34				
8. Week 8 HAM-D	10.88	5-17	0.87	0.12	0.23	0.02	0.42^*^	0.45^*^	0.21	–0.29			
9. Week 8 PHQ-9	6.79	3-10	0.93	0.21	0.28	0.12	0.25	0.42^*^	0.42^*^	–0.24	0.91		
10. Week 8 CSQ^f^	27.58	25.5-30.5	0.93	0.31	0.19	0.31	–0.24	–0.33	0.27	0.33	–0.42^*^	–0.39	
11. Time spent practicing off the app (min)	35.02	22.5-48	N/A	0.31	–0.24	0.32	–0.15	–0.17	–0.13	0.04	–0.09	–0.16	0.31

^a^IQR presented as 25th and 75th percentiles in the data.

^b^ω_t_: McDonald Omega Total, a measure of internal reliability for scale scores.

^c^N/A: not applicable.

^d^HAM-D: Hamilton Depression Rating Scale.

^e^PHQ-9: Patient Health Questionnaire-9.

^f^CSQ: Client Satisfaction Questionnaire.

^*^P<.05.

^***^P<.001.

### About Mindset

The Mindset app delivers core CBT for MDD psychoeducation and skills practice organized into 8 modules and designed to be used with therapist support over 8 weeks. See Table 3 for a summary of app content. Treatment includes psychoeducation, cognitive restructuring and core beliefs, behavioral activation, mindfulness, and relapse prevention [27,30,31]. Modules, and activities within each module, unlock one at a time after users have either completed the requisite didactic material (eg, users can practice “catching” cognitive distortions after they learn about what cognitive distortions are) or completed sufficient practice (eg, users begin scheduling weekly activities after monitoring their habits and associated mood for 1 week). This pacing is intended to reinforce regular practice and allow skills to build. Therapists supported participants’ progress in the app-led treatment through 8 weekly video-conferencing appointments (16-25 minutes each) and were available via asynchronous in-app secure messaging. Sessions were intended to monitor risk (eg, the emergence of suicidal ideation or adverse events), enhance motivation, clarify and practice the skills learned via the Mindset app, and problem-solve treatment barriers. Therapists were licensed, doctoral-level clinicians with training in CBT for MDD. They had access to a therapist dashboard that displayed patient progress in the app. Weekly supervision from the principal investigator (expert in CBT) was also provided. Therapists were instructed to focus on whatever content patients had engaged with in the app and not to introduce additional therapeutic skills or modalities. Fidelity was monitored in weekly supervision, via therapist self-checks included within session records, and via adherence ratings (ie, the degree to which forbidden content was introduced). Notably, 100% (40/40) of rated session tapes (40/212, 19%) were rated to be completely adherent. Participants attended an average of 7.6 (SD 1.5) of 8 available therapist appointments and sent a median of 0 (IQR 0-4) between session messages to their therapist through the app.

**Table 3:** Mindset for depression content.

Content	Module	Activities
Psychoeducation on major depressive disorder and cognitive behavioral therapy	1	Read and watch videos of didactic material
Cognitive restructuring	1	Read didactic material Practice identifying cognitive distortions Practice constructing alternative thoughts
Understanding the relationship between behavior and mood	2	Read didactic material Record activities and associated mood
Behavioral activation	3-8	Read didactic material Schedule activities Record activities and associated mood
Mindfulness	4-6	Read and watch videos of didactic material Listen to guided mindfulness exercises
Modifying core beliefs and building self-esteem	7	Read didactic material Practice identifying and challenging negative core beliefs
Relapse prevention	8	Read didactic material Implement strategies to consolidate skills and maintain gains

### Measures

#### Overview

Independent evaluators (ie, Master’s and doctoral-level clinicians) assessed participants for study eligibility at baseline using the MINI (Mini International Neuropsychiatric Interview, version 7.02 [32]), which is a semistructured diagnostic assessment of the *DSM-5* psychiatric disorders.

#### Depression Symptom Measures

To measure clinician-rated depression symptom severity, trained independent evaluators administered the Hamilton Depression Rating Scale (HAM-D; [33]). The HAM-D consists of 21 items rated on a mixture of 3- and 5-point Likert scales, with the total score equaling the sum of the first 17 items. Scores can range from 0 to 52, with higher scores indicating greater depression severity. The HAM-D has demonstrated substantial reliability and validity as a measure of depression severity across many populations (see [34] for review).

To measure self-reported depression symptom severity, participants completed the PHQ-9. The PHQ-9 is a widely validated measure (see [35]) consisting of 9 Likert scale items ranging from 0 (not at all) to 3 (every day) that map onto the *DSM-5* symptom criteria of MDD. Higher scores indicate greater depression severity. The HAM-D and PHQ-9 were both administered at baseline and posttreatment (week 8) to establish change in depression severity throughout treatment.

#### Mindset Belief Measures

Participants’ beliefs about the credibility of Mindset and their degree of optimism regarding the treatment having a positive effect on them were measured at baseline using only the self-report Credibility/Expectancy Questionnaire (CEQ; [36]). The CEQ has good psychometric properties [36] and includes 2 subscales that can range from 3 to 27. Higher scores correspond to higher treatment credibility and higher outcome expectancy, respectively. Participants’ satisfaction with the Mindset intervention was assessed using the 8-item Client Satisfaction Questionnaire (CSQ; [37]) at week 8 only. This self-report questionnaire has excellent psychometric properties [37]. Scores on the CSQ can range from 8 to 32, with higher scores indicating greater satisfaction.

Internal reliability for all scale measures in the present sample is provided as McDonald Omega (ω_t_) values in Table 2.

#### Mindset App Use Measures

The Mindset app passively logged every user interaction concerning the date and time that it occurred, what element of the Mindset app was opened (eg, home screen, library, or exercise), what type of action was automatically registered as occurring (eg, app opened, app paused, or exercise complete), the specific type of app content engaged with, and device information (eg, operating platform). The app also automatically logged the date that each participant unlocked each intervention module. We measured app use by counting the number of app interactions that each participant initiated throughout the entire intervention. We further identified how many overall app interactions were to app content from previous modules and how many were to app content associated with the module they were currently assigned to complete. We obtained these app interaction count variables according to the steps described in the Data Analysis section. Participants were additionally asked to answer 1 question at week 8 to capture their perceived treatment use off the app. Specifically, participants were asked how much time they spent practicing treatment skills off the app. Responses were considered in the number of minutes in integer format. More self-reported time spent practicing on and off the app corresponds to greater treatment use.

### Data Analysis

#### Identifying App Interactions

To quantify repeated engagement with app-based content, we identified each time a participant opened a unit of content (eg, video about the nature of depression, cognitive restructuring practice, or mindfulness audio) during the 8 weeks of their active treatment. This action was counted as long as the content or app was not subsequently closed in fewer than 5 seconds. We set this 5-second threshold a priori to remove instances when a person spent so little time on the content page that we could reasonably assume that they had clicked into the page without meaningfully engaging with it (while not being so restrictive as to remove potentially meaningful interactions when a participant may have been briefly reminding themselves of a principle taught in an earlier exercise). We consulted with the app developers who confirmed that there was no other way to identify errant app openings than to set a time threshold. An exercise did not have to be completed to be counted (eg, repeating 3 minutes of a 5-minute mindfulness audio or using some, but not all, of the challenging questions within a cognitive restructuring exercise). We also identified all logging behaviors (ie, scheduling activities, recording completed activities, and associated mood), counting only those that had not been submitted within 30 minutes of other logged content of the same type. We set this 30-minute threshold a priori to address logging bursts (eg, a participant who scheduled 26 activities in 1 sitting). From our perspective, a logging burst does not represent many unique interactions; returning to log throughout the day, by contrast, suggests separate attempts to integrate the app into daily life. As such, a participant who logged 26 activities within 30 minutes would be given credit for 1 activity scheduling interaction (not 26). We chose to set this threshold to 30 minutes to be conservative after comparing the number of removed surveys using candidate thresholds of 10 minutes, 20 minutes, and 30 minutes. These sensitivity tests found that the 30-minute threshold only removed an additional 31 observations than the 10-minute threshold. We elected to use the 30-minute threshold before running analyses. We refer to these retained actions as “interactions” moving forward.

#### Overall Use

We first computed the overall and per-person total number of interactions across this study (8-week treatment) and the distribution across modules. To describe between-person differences in the frequency of app interactions, we calculated the median number of interactions logged by each participant, as well as the IQR (presented as the 25th and 75th percentiles in the data).

#### Within-Module Use

We next examined the number and proportion of interactions that occurred within each treatment module during a participant’s progression through the treatment (eg, completing a module 1 cognitive restructuring exercise while in the module 1 treatment stage; listening to a module 4 guided mindful breathing exercise while in the module 4 treatment stage).

#### Between-Module Use

We then considered the number and proportion of interactions for which a participant “revisited” prior content within a module different from the stage they were currently in (eg, completing a module 1 cognitive restructuring exercise while in the module 3 treatment stage and listening to a module 4 guided mindful breathing exercise while in the module 8 treatment stage). For modules that were revisited, we examined which activities within that module were most frequently revisited.

#### Exploratory Analyses

Exploratory analyses were conducted in R (version 4.3.3, R Foundation). First, to explore which baseline characteristics (user age, PHQ-9, HAM-D, CEQ-credibility, or CEQ-expectancy) and treatment-focused self-report features (CSQ or perceived treatment use) predicted number of app interactions (overall, within-module, and between-module), we conducted a series of univariate quasi-Poisson regression models with robust SEs. Quasi-Poisson models were fit using the “glm” function in the base R *stats* package. Robust SEs were implemented using the *sandwich* package [38,39]. The number of overall interactions, within-module interactions, and between-module interactions were treated as dependent variables in separate models. Only 1 baseline or treatment-focused predictor variable was included in each model. This resulted in 21 total models. All predictors were grand mean-centered, apart from perceived treatment use, which was log-transformed due to its right-skewed distribution. We fit these count models with a quasi-Poisson distribution given our interaction counts evidenced overdispersion on a small sample, rendering Poisson regression and negative binomial models less appropriate, respectively. We used a robust SE estimating procedure given our small sample.

To explore whether different app use patterns predicted better treatment outcomes on PHQ-9 and HAM-D measures, we conducted a series of univariate residual change score models using robust linear regression. Specifically, week 8 PHQ-9 and week 8 HAM-D were treated as dependent variables in separate models. Baseline PHQ-9 and baseline HAM-D (both grand mean centered) were entered as independent variables in their respective models, along with the specific app use count variable under investigation. This resulted in 6 additional models. These models were fit with the “rlm” function from the *MASS* package [40]. Robust *P* values were obtained for all models with the *repmod* package [41]. We fit these models with robust methods to relax model assumptions to retain all participants in this limited sample; robust models are better able to handle outliers without biasing estimates, allowing for all participants’ data in this present sample to be included.

To reduce the risk of type I error, we applied a Benjamini and Hochberg false discovery rate (FDR) correction to all models using the “p.adjust” function in the base R *stats* package.

## Results

### Overview of Previously Reported Intervention Outcomes

A total of 24 of the 26 participants included in the analyses completed the 8-week trial. A detailed summary of app development and clinical outcomes is reported in the study by Wilhelm et al [27]; key findings are shared here. As described in the primary trial paper [27], participants self-reported using the app or practicing CBT skills (on or off the app) for a median of 50 (IQR 30-60; week 4) or 60 (IQR 30-90; week 8) minutes per week; participants accessed the app on an average of 36.8 (SD 10) days. Participants’ depression symptoms (HAM-D scores) decreased from, on average, moderate severity (mean 19.1, SD 5) at baseline to mild severity at week 8 (mean 10.8, SD 6.1; g*_ave_*=1.47; *P*<.001). By week 8, a total of 13 participants were classified as responders (HAM-D score reduction ≥50%) and 10 as in remission (HAM-D score ≤7). Further, 18 (69%) participants completed all 8 modules during the 8-week treatment period, 3 (11%) completed 6 modules, 3 (11%) completed 5 modules, 1 (3%) completed 2 modules, and 1 (3%) did not complete any modules.

### Overall Use

**Figure 1:**
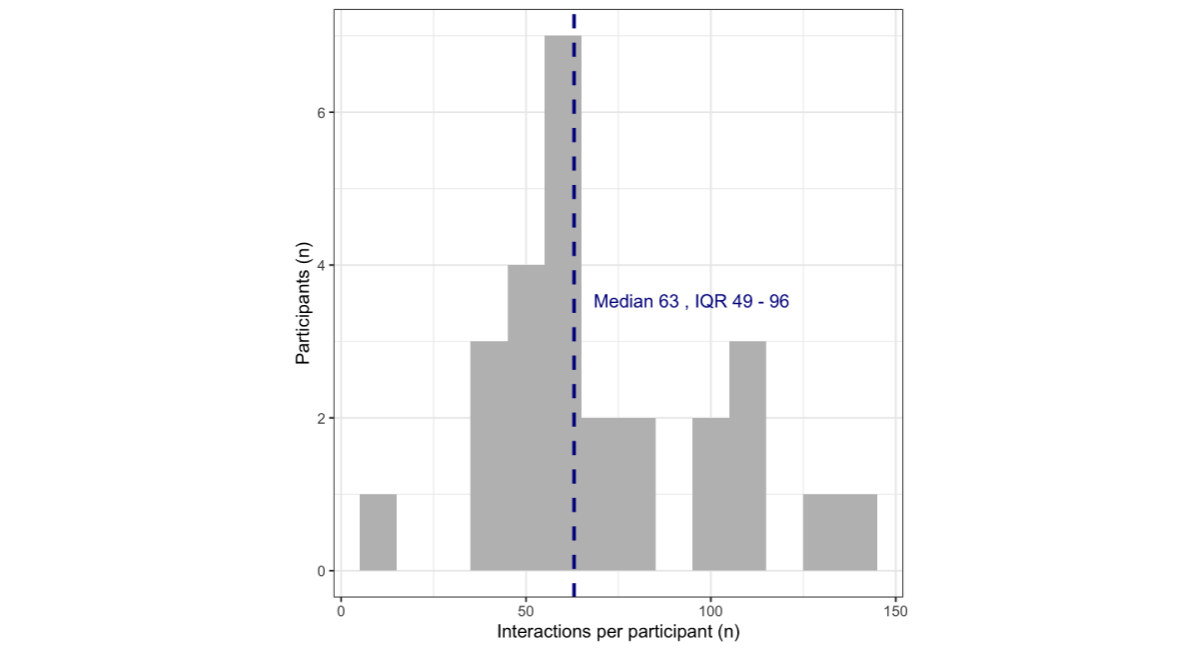
Histogram of person-level total interactions with the Mindset app. The dashed vertical line represents the median value of interactions.

Across the 26 participants with available app use data, we retained 1860 interactions across learning activities (n=254 interactions; didactic material presented via readings or videos), practice activities (n=500 interactions; active skills practice, eg, restructuring a thought or mindfulness practice), and scheduling activities and tracking associated mood (n=1106 interactions). The highest proportion of interactions occurred in modules 1 and 2 and interaction rates declined modestly across subsequent modules: 339 (18.2%) in module 1, 327 (17.6%) in module 2, 220 (11.8%) in module 3, 230 (12.4%) in module 4, 199 (10.7%) in module 5, 175 (9.4%) in module 6, 213 (11.5%) in module 7, and 157 (8.4%) in module 8. At the participant level, the number of unique interactions ranged from 8 (the participant who dropped within the first week and before completing module 1) to 144 (median 63, IQR 49-96). Note that the minimum number of interactions required to complete the full program is 62. See Figure 1 for a distribution of participant-level interactions.

### Within-Module Use

**Figure 2:**
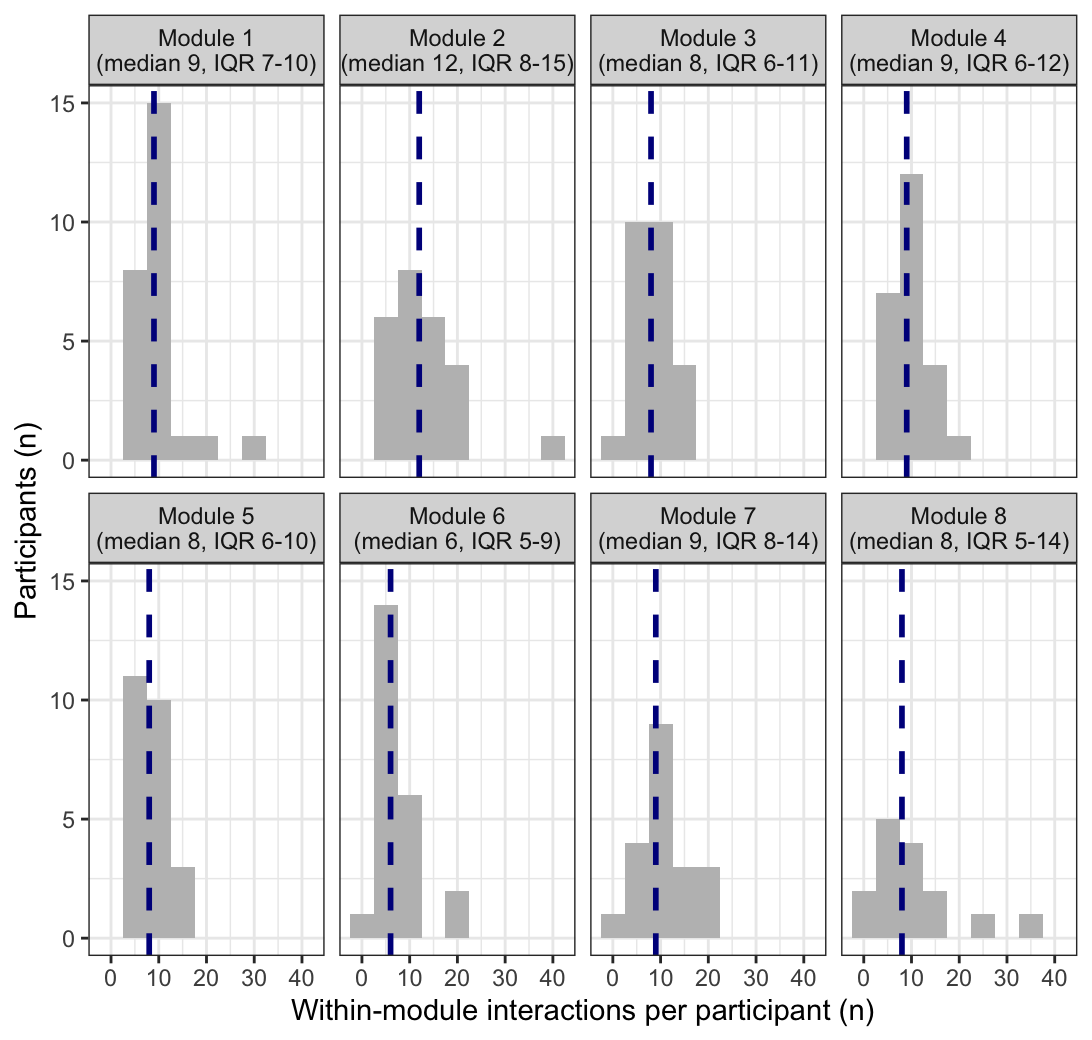
Histogram of within-module interactions with the Mindset app by treatment module. Dashed vertical lines represent the median value of within-module interactions for each module. Module 1: psychoeducation and cognitive restructuring; Module 2: behavioral monitoring; Module 3: SMART goals and behavioral activation; Module 4: mindful breathing and behavioral activation; Module 5: grounding and behavioral activation; Module 6: letting go of thoughts and behavioral activation; Module 7: core beliefs and self-esteem; Module 8: relapse prevention; SMART: goal-setting framework that helps people achieve their goals by making them specific, measurable, achievable, relevant, and time-bound.

**Figure 3:**
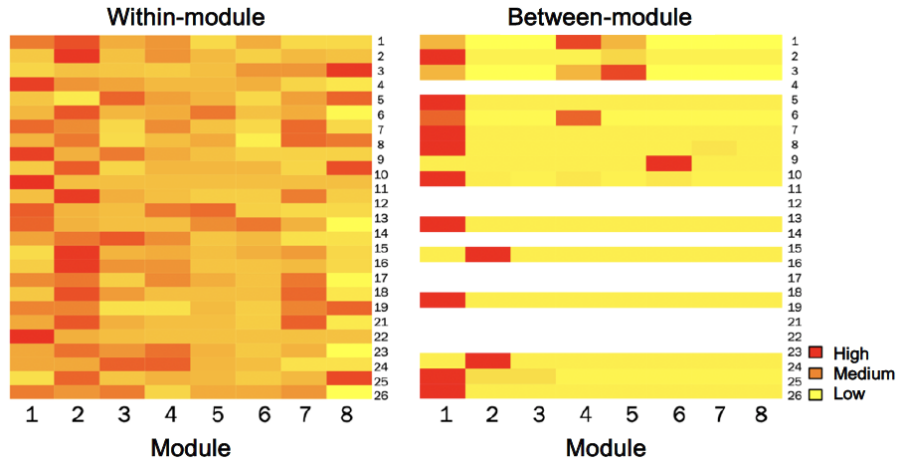
Participant-level frequency of within- and between-module interactions. Module 1: psychoeducation and cognitive restructuring; Module 2: behavioral monitoring; Module 3: SMART goals and behavioral activation; Module 4: mindful breathing and behavioral activation; Module 5: grounding and behavioral activation; Module 6: letting go of thoughts and behavioral activation; Module 7: core beliefs and self-esteem; Module 8: relapse prevention; SMART: goal-setting framework that helps people achieve their goals by making them specific, measurable, achievable, relevant, and time-bound.

Out of all 1860 interactions retained, 94.3% (n=1754) comprised activities within a participant’s current module. At the participant level, within-module interactions also ranged from 8 to 144 (median 60.50, IQR 49-80). Figure 2 visualizes the distribution of within-module interactions across treatment modules. Most participants made more interactions than were necessary across all modules and they tended to return to all types of available content (eg, revisiting a psychoeducational video a few times and logging more activities than necessary to progress). Moreover, there were no “clear winners” for which specific pieces of app content participants tended to use at higher than required rates. The one exception is concerning activity logging; for example, 1 participant entered 30 logs throughout module 8, even after log bursts were removed. This also explains the right skew to the distributions seen in some modules, which otherwise tended to be relatively tightly clustered. See Figure 3 for a visualization of participant-level behavior.

### Between-Module Use

**Figure 4:**
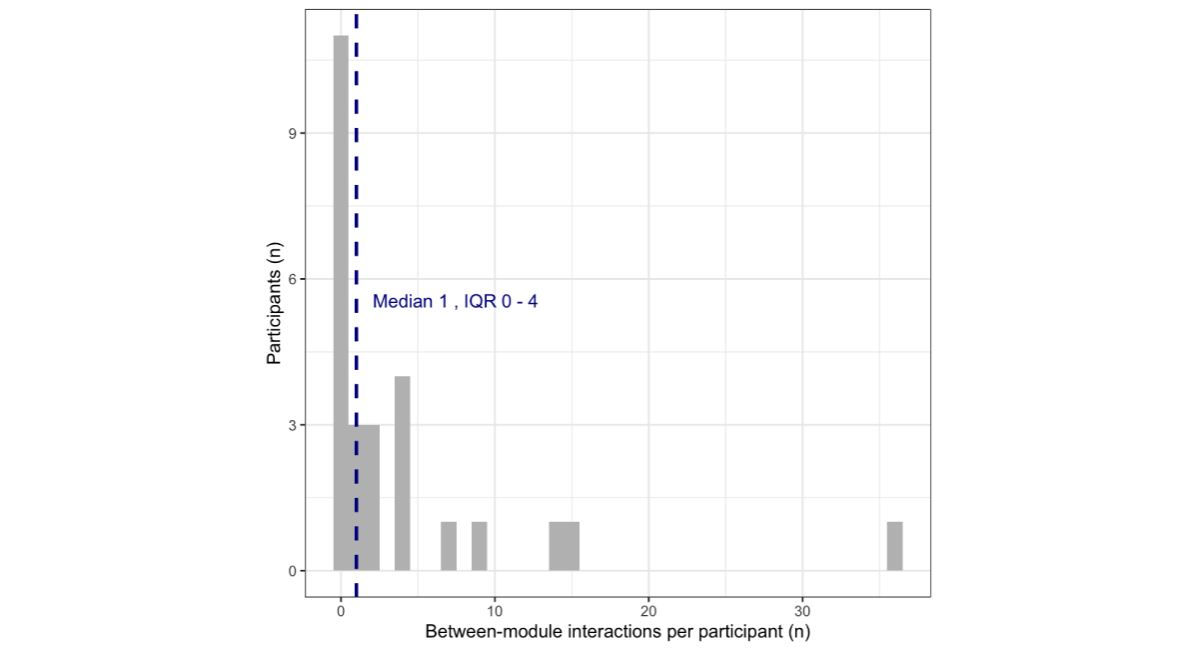
Histogram of person-level between-module interactions with the Mindset app. The dashed vertical line represents the median value of interactions.

**Figure 5:**
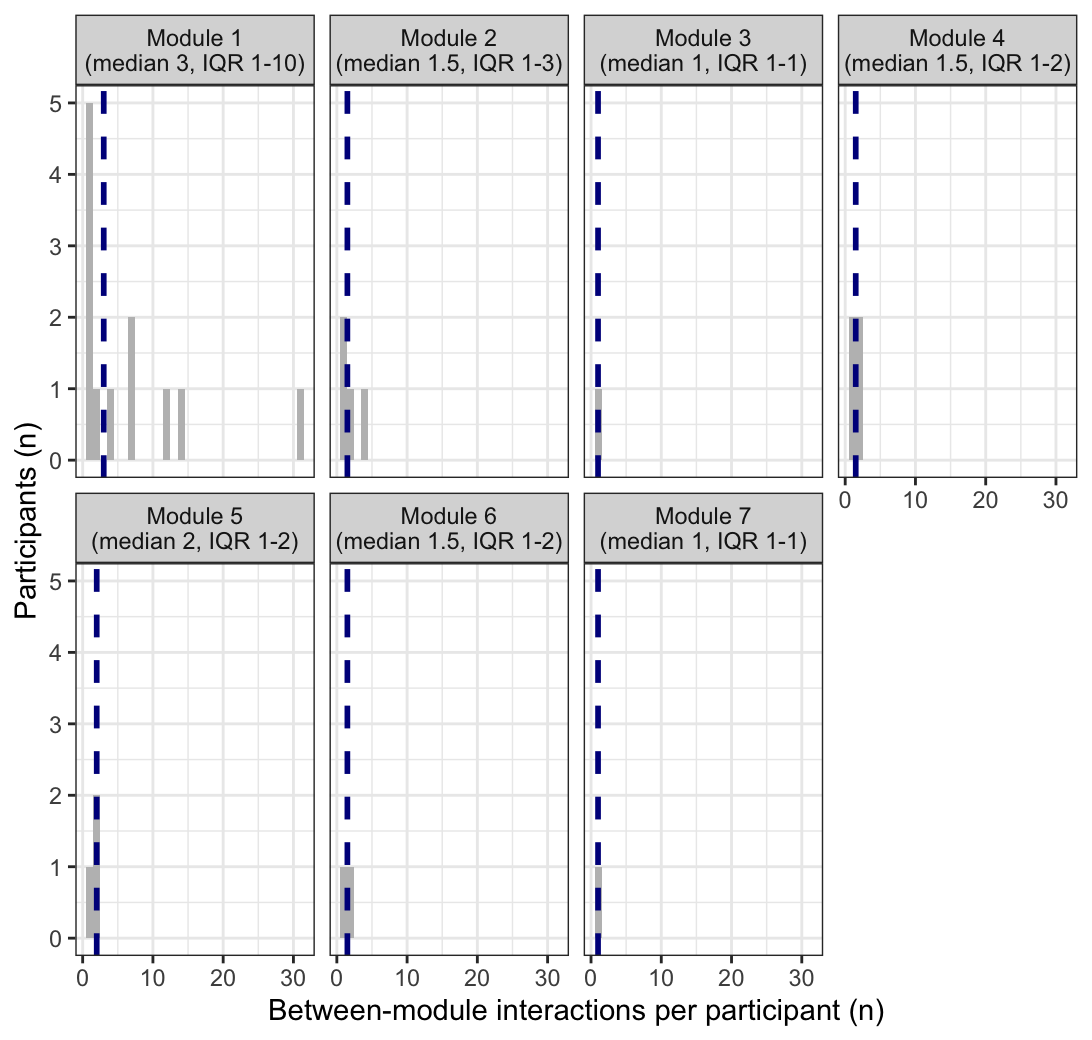
Histogram of between-module interactions with the Mindset app by treatment module. Dashed vertical lines represent the median value of between-module interactions (ie, “revisits”) for each module. Module 8 is not represented because, by definition, module 8 could not be revisited.

A total of 106 (5.7%) interactions comprised activities that met our a priori definition of “revisiting” a previous module. At the participant level, between-module interactions ranged from 0 to 36 (median 1, IQR 0-4). Figure 4 visualizes the distribution of person-level between-module interactions. Although recommended, between-module use was not required to progress through treatment content; in practice, between-module use was very rare in these data. Further, 11 (42.31%) participants never revisited any previous module. Of the between-module uses observed, over three-quarters were to a module 1-associated activity (82 out of 106, which were registered by 12 participants). Of the 82 revisits to module 1, a total of 12 (14%) were to psychoeducational content (10 revisits to general CBT overview, 2 revisits to psychoeducation on the nature of depression), 33 (40%) were to a practice exercise accessed through the module 1 page (eg, “catch the thinking trap” and “evaluating your thoughts”), and 37 (45%) were to the “check in with your thoughts” practice tool accessed through the library. The higher likelihood of returning to module 1 is reflected in Figure 5, which visualizes the distribution of between-module interactions across treatment modules. Participant-level behaviors are visualized in Figure 3.

### Exploratory Analyses

Spearman correlations between individuals’ number of overall, within-, and between-module interactions and baseline (HAM-D, PHQ-9, CEQ-Credibility, and CEQ-Expectancy) and end-of-treatment (HAM-D, PHQ-9, CSQ, and perceived treatment use) features are provided in Table 2. Although the total number of interactions and the number of within-module interactions were significantly positively correlated with each other, neither of these 2 app use features were significantly associated with any of the clinical features examined. The number of between-module interactions was not significantly correlated with overall or within-module interactions, nor with any clinical or treatment-focused self-reported feature.

Analyses exploring potential predictors of treatment use patterns found that age was significantly positively associated with the number of between-module interactions (*b*=0.07, SE 0.03, *z*=2.70, 95% CI 0.011-0.129; *P=*.007, rate ratio=1.07); baseline treatment credibility was significantly positively associated with the number of between-module interactions (*b*=0.17, SE 0.09, *z*=2.01, 95% CI 0.0014-0.34; *P=*.04, rate ratio=1.19); and week 8 treatment satisfaction was significantly positively associated with the number of overall (*b*=0.05, SE 0.02, *z*=2.27, 95% CI 0.01-0.09; *P=*.02, rate ratio=1.05) and within-module app interactions (*b*=0.04, SE 0.02, *z*=1.97, 95% CI 0.001-0.08; *P=*.049, rate ratio=1.04). Rate ratios can be used to interpret effect sizes within count models. For example, the rate ratio of 1.07 above suggests that, holding all other predictors constant, for each 1 unit increase in age the expected count of within-module logins increases by a factor of 1.07. Residual change score models found that the number of between-module interactions was significantly positively associated with week 8 PHQ-9 scores, even after accounting for baseline PHQ-9 scores (*b*=0.23, SE 0.11, *t*=2.10, 95% CI 0.014-0.446; *P=*.049). No other explored effects were significant at an α level of .05. None of the above effects survived FDR correction.

## Discussion

### Principal Findings

In this study, we quantified how participants engaged with didactic content and guided skills practice within and across treatment modules of Mindset, an 8-week therapist-guided CBT smartphone app for MDD. Our goal was to investigate to what degree people repeated and revisited content, and whether more repetition promoted better treatment outcomes. We found that 18 (69%) individuals completed all content within the app and 14 (78%; 53% of the total sample) of those individuals engaged with content more times than was required to complete the treatment protocol. The number of interactions logged varied widely across the sample (median 63, IQR 49-96), and only 106 (5.7%) of those interactions were to revisit content from a previous module. Before FDR correction, exploratory analyses found that older participants tended to log more app revisits; participants who saw the treatment as more credible at baseline tended to log more app revisits; participants who were more satisfied with the treatment at week 8 tended to log more overall and within module app interactions; and participants with worse self-reported depression symptoms at week 8, after controlling for baseline self-reported depression symptoms, tended to log more app revisits.

Despite the ease of access, mental health app use rates are generally low in clinical trials for depression [13,42] and are even worse outside of research contexts [13,43]. Yet, there remains meaningful variability. For example, a recent scoping review of digital mental health interventions for depression reported attrition rates across 11 studies ranging from 7% to 85.6% and only 50% or fewer participants completed their given digital intervention as intended [16]. This suggests that important differences in user characteristics, app content, and app design may be differentially associated with engagement. The present data extend this literature by not only reporting completion rates but also by highlighting the degree to which some users repeated content (ie, beyond typical criteria for adherence or completion). Indeed, approximately half of the present sample engaged with the app more times than was strictly necessary—even with regular appointments with a therapist, which offers modest support for the hope that providing treatment material at users’ fingertips will translate into frequent, personally driven use of that material. That said, the number of overall interactions with the app was unrelated to any baseline or end-of-treatment variables. This further motivates a more nuanced look at how patients use available content and whether they naturally gravitate toward the types of content and cadence of app use that are most likely to be beneficial to them [44].

Although just over half of the users in this study reviewed the content of all types more than was required (eg, reread psychoeducation or logged more than the minimum number of activities required to progress in the app), 11 (42%) participants did not revisit content from previous modules at all. Moreover, 10 of the 15 participants who did revisit a previous module did so fewer than 5 times. Participants instead tended to focus on whatever content was delivered within a given module. This suggests that most users may rely on the program to suggest the type of work that would be most beneficial for them at a given point in treatment. Thus, if developers intend for users to review old content throughout a trial, the typical user may require explicit reminders to return to previous content, repetition built into future modules, or a feature allowing for bookmarked exercises to be highlighted and easily found. This also presents a clear opportunity for user-centered and co-design methods as well as more effective collaboration with designers [45]. This would allow greater insight into how users navigate digital mental health tools and what product modifications could have the greatest impact.

Of the participants who returned to previous app content, they most often revisited foundational content such as psychoeducation (regarding depression and the CBT model) and cognitive skills (eg, identifying and challenging maladaptive thoughts) in particular. This may simply be due to their being introduced earliest in treatment and thus more likely to be forgotten later on, necessitating a revisit. This pattern could also align with past work on user preference; in a survey-based study interviewing smartphone owners (n=119) with depression, anxiety, or both, 64.7% of respondents endorsed interest in psychoeducation as a specific feature of a mental health app and 72.3% were interested in a module on cognitive restructuring [46]. Mastering psychoeducational content may be advantageous given known associations between health literacy—or understanding one’s condition—and treatment options, adherence, and outcomes [47,48]. Many participants in this study had never received treatment for MDD before and could thus have relied more heavily on these components to gain insight and to better understand their progress. Interestingly, although there is a lack of research comparing engagement with psychoeducation or cognitive skills versus other content within the same app, studies comparing engagement across mental health apps suggest that individuals typically engage with these elements less than others; for example, apps using psychoeducation as the primary tool have lower rates of engagement in terms of daily minutes when compared to apps with other tools, such as mindfulness or meditation and peer support [13]. This again, however, highlights the importance of examining what users are engaging with; it may simply require fewer minutes to consume brief psychoeducational material than to complete a 10-minute mindfulness exercise. Similarly, stand-alone cognitive apps seem to have sharper drop-offs than behavioral activation-focused programs. Taken together, present results highlight the need to consider the interaction between treatment components; for instance, individuals may be more likely to return to foundational content when it is integrated with more actionable accompanying skills such as behavioral activation. Helping users to understand the personal relevance of the content and the anticipated effect on their mood may increase engagement. Again, bringing users into this process is essential to truly understand the observed patterns, their impact on outcomes, and the desired program changes.

Before FDR correction, baseline treatment credibility was positively associated with revisiting prior modules. In other words, participants who entered this study with more positive views of CBT or mHealth approaches were more likely to revisit material during treatment. This finding may suggest that participants with more initially positive views of Mindset were more motivated to return to foundational skills to glean more from treatment. Yet, revisits were overall very infrequent, and using the app more overall—within-module in particular and again before FDR correction—was ultimately associated with more satisfaction with the program. As causality cannot be determined presently, it is possible that individuals who found the program less helpful or personally relevant chose to engage less; it is also possible that engaging more—particularly with the activities that the program assigned each week—led participants to feel that they were benefiting or connecting more with the materials. Both app use and satisfaction could also be explained by third variables, such as perceived fit (ie, how well the treatment aligns with a person’s symptoms and preferences) or motivation. Replication in a larger sample is required to tease apart this association.

Surprisingly though, more revisits in the present sample were associated with more severe end-of-treatment self-reported depressive symptoms, after controlling for pretreatment self-reported depressive symptoms. Although we expected more frequent revisits to boost treatment efficacy, it could be that participants with relatively elevated symptoms after 8 weeks had good insight into their need for extra support throughout the intervention, which prompted them to return to foundational materials more often. This pattern could also be because people with higher levels of depression may have had greater difficulty remembering or required more repetition due to the cognitive symptoms of MDD (eg, poor concentration), felt less confident in their knowledge about the foundational psychoeducational content, or may have felt frustrated by lack of progress; as such, their revisits may have represented lesser mastery of the information or cycling back through the material to see if anything critical may have been missed. Yet another potential explanation is that stronger clinical response is associated with greater independent skills use off the app (ie, enactment in daily life); thus, sustained reliance on the app could suggest lesser comprehension of skills or difficulty integrating skills into daily life. However, we did not see any association between the perceived number of minutes reportedly spent practicing off-app and any clinical or app use feature in the present sample.

That said, our finding is interestingly misaligned with another trial of app-based CBT for MDD, which found that participants with better treatment response completed more in-app cognitive restructuring and behavioral activation experiments than peers with lesser response [25]. Of note, participants in the trial were supported by prescribing physicians, rather than CBT therapists, and with variable frequency, making it more challenging to directly compare the studies. It is also possible that our finding is an artifact of a small sample with low variance—again, few people revisited any content, and those who did usually did so infrequently—or the availability of a CBT therapist for skills review. Moreover, this effect did not survive FDR correction, so it is similarly possible our finding is a false positive in need of replication in a larger sample. However, this potential discrepancy highlights broader questions in the field; evidence for how best to capture the use of digital treatments (guided and self-guided; for depression and other targets) and whether use patterns relate to outcomes remains mixed [18]. While some studies have found significant positive associations between engagement and outcomes [49], others have not [50]. Such heterogeneity encourages moving away from one-size-fits-all approaches; recommending the same dose or pace for all patients is likely inefficient and is possibly reducing overall efficacy.

Indeed, researchers have begun to favor “effective engagement” over simply more engagement [51] and highlighted the need for greater integration of evidence-based engagement strategies in addition to cognitive behavioral ones [52]. Users would likely benefit from more personalized recommendations that offer greater insight into their own behaviors. Increased insight facilitates deeper user engagement with digital mental health interventions [53] and is itself associated with better treatment outcomes [54]. One example of personalization is providing ongoing feedback on progress to bolster motivation and guide users to where they could benefit from additional review or continued practice [55]. Additionally, individual characteristics may be informative; in this sample, older participants were more likely to revisit content from earlier modules than their younger counterparts. Although this requires replication in a larger sample this pattern could reflect different learning needs. Tailoring interventions to each user’s individual needs has been shown to reduce attrition [42,56] and lead to improved mental health outcomes [57,58]. Moreover, research suggests that too frequent engagement with certain aspects of mental health apps may inadvertently lead to fatigue and could thus contribute to worse outcomes [50]. Thus, continuing to examine what people are engaging with and how, rather than mere frequency of use, is critical for optimizing digital interventions. In particular, it will be critical to partner with users and other stakeholders to better clarify, operationalize, and evaluate the malleability of subjective aspects of engagement that appear to have an outsized impact on treatment response [59].

### Limitations

First, our sample size from this pilot trial is small and has limited demographic heterogeneity (ie, n=21, 80.8% identified as women; n=21, 80.8% identified as non-Hispanic; n=18, 69.2% identified as White); as such, demographics could not be examined as predictors of app interactions or outcomes. Moreover, no effects remained significant after the FDR correction. The present data were powered as an acceptability and feasibility intervention pilot study without respect to app use patterns, potentially rendering these analyses underpowered. Replication in larger, more generalizable samples is therefore needed. However, these data highlight that heterogeneity in-app use, even among a small sample, is high and warrants continued investigation. Second, given the design of the Mindset app, we were only able to consider the use of “pull” components, or content that is available for access in the app and with which the user must initiate engagement. Digital therapies more broadly are also able to deliver content via “push” components, or content sent to users with the hope of providing treatment when it is most likely to be received and effective (eg, “just-in-time” interventions). They do not require participants to be aware of their need for support or even remember that the tool exists [60]. It is possible that participants would have revisited content more or in different ways had there been pushed suggestions or activities, and engagement with push components may relate differently to demographic or clinical variables than the pull interactions we investigated. Similarly, it will also be important to consider Mindset app use in the absence of therapist check-ins that were available to all patients in this study (ie, self-guided app-based treatment). We were also only able to objectively capture “on-app” behaviors when it is likely that “off-app” behaviors are also powerful for symptom change. Of course, a foundational assumption is that more frequent skills review and app use contribute to better outcomes by facilitating off-app change; although perceived time using skills “off-app” was not associated with app interaction patterns, it remains possible that more rigorous assessment of how often participants were thinking about or implementing CBT skills without the aid of the Mindset app or the quality of the “off-app” work would reveal different associations (eg, daily diary method rather than end-of-treatment measure). Indeed, it remains plausible that higher responders in this trial were going “off-app” more and earlier than others. As with any effort to quantify behavior, we necessarily made assumptions about users and what constituted a meaningful interaction with CBT content (eg, <5 s indicated an erroneous action; multiple logs within 30 minutes indicated one practice session). Addressing all of these limitations and doing so in a larger and more diverse sample is an important next step. Finally, treatment adherence in this study was high and attrition was very low compared to the typical smartphone CBT; a larger, real-world trial would likely provide greater variance and thus further insights.

### Conclusions

Some degree of treatment use is important for outcomes [61]. However, it is oversimplified, if not incorrect, to argue that more use is necessarily better. Instead, more nuanced conceptualization and measurement are needed and will hopefully lead to more personalized recommendations for app use [19]. Indeed, whereas 1 person may benefit from frequent reminders to be mindful or require repeated exposure to a concept for it to resonate, others may respond quickly, benefit most from fewer massed exposures, or benefit from content unlocking at a faster or slower rate than others. Past work on engagement has suggested that module completion, rather than volume of use, is a more clinically relevant metric of engagement; this recommendation aligns with our finding that repetition was not associated with better outcomes [18]. Minimum requirements or recommendations for engagement were thoughtfully developed and may on average be sufficient. At the same time, response and remission rates in this trial—similar to other trials—could be much stronger. These data underscore the high degree of heterogeneity that exists in users’ app use patterns. More work such as this—particularly in larger and more representative samples—which aims to explain this heterogeneity in what users engage with and how they do so is essential for driving more flexible therapies, more personalized dosing and sequencing, and better outcomes.

## Abbreviations

CBT: cognitive behavioral therapy
CEQ: Credibility/Expectancy Questionnaire
CSQ: Client Satisfaction Questionnaire
DSM-5: Diagnostic and Statistical Manual of Mental Disorders (Fifth Edition)
FDR: false discovery rate
HAM-D: Hamilton Depression Rating Scale
MDD: major depressive disorder
Mindset: Mindset for Depression
MINI: Mini International Neuropsychiatric Interview
PHQ-9: Patient Health Questionnaire-9
